# Perspectives on the pH-Influenced Design of Chitosan–Genipin Nanogels for Cell-Targeted Delivery

**DOI:** 10.3390/pharmaceutics17070876

**Published:** 2025-07-03

**Authors:** Julieta D. Glasman, Agustina Alaimo, Cecilia Samaniego López, María Edith Farías, Romina B. Currá, Diego G. Lamas, Oscar E. Pérez

**Affiliations:** 1 Departamento de Química Biológica, Facultad de Ciencias Exactas y Naturales, Universidad de Buenos Aires, Buenos Aires C1428EGA, Argentina; jglasman@qb.fcen.uba.ar (J.D.G.); aalaimo@qb.fcen.uba.ar (A.A.); 2 Instituto de Química Biológica, Facultad de Ciencias Exactas y Naturales, Consejo Nacional de Investigaciones Científicas y Técnicas (IQUIBICEN-CONICET), Buenos Aires C1428EGA, Argentina; cecisamaniego@qo.fcen.uba.ar; 3 Departamento de Química Orgánica, Facultad de Ciencias Exactas y Naturales, Universidad de Buenos Aires, Buenos Aires C1428EGA, Argentina; 4 Grupo de Investigación en Nanopartículas y Reología de Biopolímeros Alimenticios (GINaRBA), Departamento de Tecnología, Universidad Nacional de Luján, Buenos Aires B6700ZBA, Argentina; 5 Escuela de Ciencia y Tecnología, Laboratorio de Cristalografía Aplicada, Instituto de Tecnologías Emergentes y Ciencias Aplicadas (ITECA), Universidad Nacional de San Martín—CONICET, General San Martín B1650HML, Argentina

**Keywords:** chitosan, genipin, nanogels, biocompatibility, drug delivery

## Abstract

**Background**: Chitosan (CS) crosslinked with genipin (GNP) provides a mild, non-toxic route to generate nanogels (NGs) with enhanced integrity and colloidal stability. **Objectives**: To develop and characterise CS-GNP NG as a novel platform for targeted cellular delivery, optimising design through physicochemical characterisation and biocompatibility evaluation. **Methods**: NGs were synthesised under optimised conditions by adjusting the pH of the CS solution, followed by high-intensity ultrasound (HIUS) to achieve disaggregation. Physicochemical characterisation was carried out using UV-Vis spectroscopy, FTIR, dynamic light scattering (DLS), and scanning electron microscopy (SEM). Rheological studies and SAXS analysis assessed structural properties. Biocompatibility was evaluated via MTT assay, and internalisation was monitored by fluorescence microscopy on mammalian cell lines. **Results**: NG formation was highly pH-dependent, with optimal configuration at pH 4.5, yielding stable, uniformly sized particles (~200 nm, ζ-potential +29 mV). Kinetic modelling showed a sigmoidal formation pattern, suggesting nucleation, growth, and stabilisation. FTIR confirmed covalent bonding between CS and GNP via primary amide bonds and Schiff bases. Rheology indicated pseudoplastic behaviour, and SAXS revealed a compact network formation. Biocompatibility assays confirmed non-cytotoxicity below 100 µg/mL and efficient cellular uptake. **Conclusions**: This study presents a rapid, reproducible protocol for generating colloidally stable, biocompatible NGs suitable for drug delivery.

## 1. Introduction

Nanogels (NGs) are three-dimensional, nanometric-sized entities formed by crosslinked polymeric networks that emerged as an innovative technology in the field of controlled drug delivery. NGs have the ability to encapsulate and release therapeutic compounds in a sustained manner in response to specific stimuli, such as pH or temperature changes. These properties make NGs promising tools for biomedical applications, particularly in the design of targeted therapies. Furthermore, their high colloidal stability, biocompatibility, and biodegradability reduce the risks of adverse side effects [1,2].

Chitosan (CS) is commonly derived from the partial deacetylation of chitin, a naturally abundant linear copolymer of *N*-acetyl-D-glucosamine. Chitin constitutes a major structural component in a variety of biological matrices, including the exoskeletons of marine arthropods (e.g., shrimp and crabs), the cuticular layers of insects, and the cell walls of fungi and certain other microorganisms [3]. CS stands out as a versatile biopolymer thanks to its antimicrobial properties, mucoadhesive capacity and biodegradable nature. Derived from the partial deacetylation of chitin, CS is composed of glucosamine and N-acetylglucosamine units linked by β(1–4) bonds. Its limited solubility in neutral media and its low stability restrict its direct application under physiological conditions [4,5]. However, this polysaccharide can be adequate for forming new structures via chemical crosslinking, forming more stable three-dimensional networks.

Genipin (GNP) is a natural crosslinking agent derived from the evergreen shrub *Gardenia jasminoides*. GNP has attracted attention as a promising alternative to other toxic compounds such as glutaraldehyde [6]. Notably, GNP was reported to efficiently crosslink with polysaccharides bearing primary amine groups, such as carrageenan/carboxymethyl cellulose [7], amino-grafted cellulose [8], derivatives of alginate [9], starch [10], hyaluronic acid [11,12], and CS [13]. The GNP-mediated crosslinking of these amine-bearing polysaccharides improves their mechanical strength, enzymatic stability, and resistance to aqueous environments, thereby expanding their biomedical potential [14]. In particular, GNP forms stable covalent bonds with the amino groups of CS, which results in the development of a characteristic blue coloration and red fluorescence, facilitating experimental monitoring during the nanogel (NG) formation process [15].

The crosslinking mechanism between CS and GNP, which leads to the NG generation, is highly dependent on the pH of the CS solution [16]. At pH < 6, the primary amino groups (-NH_2_) in the glucosamine units CS’s chain react nucleophilically with GNP to form amide bonds, generating dense polymeric networks of high stability. The crosslinking reaction involves two distinct steps. Firstly, the opening of the GNP ring by a nucleophilic attack carried out by the nitrogen of a primary amino group of CS. This attack typically occurs on the C-3 carbon of GNP, breaking the GNP ring and originating an intermediate that retains a transient aldehyde group (-CHO). This first step generates a covalent C-N bond and yields a heterocyclic compound. In addition, the ring opening initiates the possibility of subsequent oligomerisation or polimerisation reactions of GNP. The second step is the nucleophilic substitution of the remaining ester group in the GNP molecule, following ring opening, and leads to the formation of a bridge between two different points of the same chain or different chains of CS. As a result of these two steps, covalent crosslinks are formed between the polymer chains, leading to the formation of a three-dimensional network. At pH > 7.5, GNP self-polymerises before reacting, creating less dense but more flexible networks [17,18]. Such differences allow the design of NGs to tailor to the needs of each application [19,20].

In this context, the main objective of this contribution is to develop and to characterise CS-GNP NG as a novel platform for targeted cellular delivery, optimising their generation process with their physicochemical properties’ characterisation and biocompatibility evaluation, considering pH variations under mild and controllable conditions. To achieve this, particle size distribution, colloidal stability, ultrastructure, swelling, and intramolecular organisation were thoroughly assessed. As these NGs are intended to serve as targeted delivery systems, the study concludes with a cell-based assay to track the fate of these entities upon contact with biological material.

## 2. Materials and Methods

### 2.1. Reagents

CS with a degree of deacetylation of 91.4%, as determined by ^1^H NMR, and an average molecular weight of 192 kDa was purchased from Parafarm^®^ (Saporiti S.A.C.I.F.I.A., Buenos Aires, Argentina; Ref. # 11017A) [21]. GNP >98% purity (CAS 6902-77-8) (sc-203057A) and Trypan Blue powder (CAS 72-57-1) (sc-216028), were purchased from Santa Cruz Biotechnology, Corp. Dallas, TX, USA). Glacial acetic acid (≥99% purity) was from Merck (Darmstadt, Germany). Foetal Bovine Serum (FBS) was purchased from Internegocios S.A. (Buenos Aires, Argentina). 3-(4,5-dimethyl-thiazol-2-yl)-2,5-diphenyl-tetrazolium bromide (MTT) and 4′,6-diamidino-2-phenylindole (DAPI) were from Sigma-Aldrich Co. (St. Louis, MO, USA). Dulbecco’s Modified Eagle’s Medium (DMEM), DMEM/F-12, Trypsin-EDTA 0.5% (10×), Antibiotic–Antimycotic (penicillin, streptomycin, amphotericin B, 100×), and GlutaMAX™-I (L-alanine-L-glutamine, 100×) were all obtained from Gibco, Thermo Fisher Scientific (Waltham, MA, USA). Any other chemicals were analytical grade. All aqueous solutions used were prepared from deionised Milli-Q water (Millipore, Bedford, MA, USA).

### 2.2. Generation of CS-GNP NG

CS-GNP NG were prepared by covalent gelation in aqueous-ethanolic medium, adapted from previous protocols [20,22,23,24,25]. Firstly, CS was dissolved at a concentration of 0.3% *w*/*v* in 1% *v*/*v* acetic acid. The dissolution was carried out under constant stirring (300 rpm) at room temperature (~22–25 °C) for 24 h in hermetically sealed glass containers to avoid evaporation and ensuring complete hydration of CS [23]. To compare different pH values for CS solutions (3.6, 4.5, 5.5), 0.1 M NaOH was added dropwise, and pH values were monitored with a PHS-3E pH Meter (Arcano, Buenos Aires, Argentina). The adjustment was performed slowly to avoid CS precipitation and ensure a homogeneous environment for the crosslinking reaction. In parallel, a GNP solution in absolute ethanol was prepared at a concentration previously defined according to the CS:GNP ratio indicated in Table 1. The GNP solution was protected from light by wrapping the container in aluminium foil and stored at −20 °C until use, in order to preserve its chemical stability and avoid premature reactions [24,25].

For NG generation, 0.6 mL of the CS solution (previously adjusted to the corresponding pH) was placed in a 1.5 mL Eppendorf^®^ tube (Eppendorf, Hamburg, Germany). Then, 0.4 mL of the GNP solution was added dropwise using an automatic micropipette, while the mixture was kept under constant stirring at 350 rpm in a Thermomixer Comfort (Eppendorf, Hamburg, Germany). This slow addition promotes a more controlled and homogeneous crosslinking between the amine groups of CS and the reactive ester groups of GNP. The mixtures were incubated at 37 °C for 24 h in total darkness, maintaining constant agitation at 350 rpm to favour the progressive formation of covalent bonds and prevent sedimentation. The development of an intense blue colour was used as a visual indicator of successful crosslinking, as this signal is associated with the formation of Schiff-type covalent bonds between the free amine groups of CS and the aldehyde groups of GNP. However, this observation was not used as conclusive evidence of NG formation, but only as a control of the reaction progress [8]. Finally, the samples were subjected to high-intensity ultrasound (HIUS) treatment to reduce particle size and disaggregate possible supramolecular structures. The procedure was performed with a direct probe Polystat sonicator (Cole-Parmer, Vernon Hills, IL, USA) operating at a frequency of 20 kHz, with a maximum output power of 750 W and 20% amplitude. Ultrasound was applied for 5 min in cycles of 15 s ON/5 s OFF to avoid overheating, keeping the tubes partially submerged in an ice bath. This step was crucial to achieve a more homogeneous size distribution and establish a stable colloidal morphology, as previously reported [20].

The resulting suspensions were stored at 4 °C, until characterisation or subsequent use, for a maximum period of 72 h to avoid degradation or secondary aggregation processes.

### 2.3. NG Formation Monitoring

#### 2.3.1. Spectral Analysis

NG generation and its temporal evolution were monitored using a PolarStar^®^ plate reader (Omega version 5.10 R2, BMG Labtech, Ortenberg, Germany) equipped with temperature control (37 °C) and orbital agitation. Aliquots of each sample (200 µL) were dispensed into clear-bottom, 96-well microplates (Greiner Bio-One, Kremsmünster, Austria). A reagent blank consisting of 1% (*v*/*v*) acetic acid: absolute ethanol, matching the solvent ratio used for polymer and crosslinker dissolution, was included on each plate. Full absorbance spectra (220–800 nm) were acquired at 0 h and 24 h for all samples as well as for single-component controls (CS or GNP) to exclude potential spectral interferences. Spectral scanning was subsequently repeated at 48, 96, and 120 h. Between readings, plates were stored at 4 °C to inhibit further reaction.

Formulations displaying the characteristic chromophore centred at ≈600 nm, indicating covalent CS-GNP crosslink formation [25,26]. Time-resolved kinetic was assessed, absorbance at 600 nm was recorded every 10 min for 24 h at 37 °C under orbital shaking and in the absence of ambient light. Independent wells containing CS-only and GNP-only solutions were run in parallel as negative controls. The resulting time courses were subsequently fitted to pseudo-first-order and sigmoidal kinetic models to quantify the crosslinking dynamics.

#### 2.3.2. Kinetics Approach

To analyse the evolution of absorbance as a function of time (*A* vs. *t*), two approaches were evaluated:

Four-Parameter Sigmoidal Kinetics with the application of Equation (1):

(1) $$A\left(t\right)=\frac{A_{max}-A_{min}}{1+e^{-k(t-{\;t}_{0})\;}},$$

where *A*(*t*) is the absorbance at 600 nm at time *t*, *A_min_* and *A_max_* represent the lower and upper limits of the absorbance, *k* is the rate constant associated with the exponential phase of the curve, and *t*_0_ is the time at which the slope was maximum (inflexion). This model captures processes with an induction phase and exponential growth before reaching a plateau.

First-Order Exponential Kinetics, which is described by Equation (2):

(2) $$\left(t\right)=A^{\infty }(1-e^{-kt})$$

where *A*^∞^ is the absorbance assumed as the asymptotic maximum value and *k* is the first-order rate constant. It describes exponential growth processes with saturation.

The goodness of fit (R^2^) and the agreement with plausible reaction mechanisms were evaluated to select the most representative kinetic model. A better fit to the first-order exponential equation suggested a process that initiated rapidly and gradually slowed down as it approached the maximum absorbance value (*A*^∞^). If the experimental data fit better with the four-parameter sigmoid equation, there was a lag phase, indicating that the formation of the NG required a nucleation time before the process accelerates (growth step) [27,28].

#### 2.3.3. Fourier-Transform Infrared (FTIR)

To verify the generation of covalent bonds between CS and GNP, FTIR spectroscopy with ATR module (FTIR-ATR Nicolet IS20, Madison, WI, USA.) was used with a resolution of 4 cm^−1^, recording 32 scans per spectrum in the range of 4000–500 cm^−1^. The NG samples were previously freeze-dried, then placed directly on the ATR crystal. Control measurements were performed using CS and GNP individually under the same experimental conditions [29].

### 2.4. NG Characterisation

#### 2.4.1. Particle Size Distribution and ζ-Potential

The intensity vs. d.nm profiles and polydispersity index (PDI) of each NG sample were determined by Dynamic Light Scattering (DLS), using a Litesizer 500 (Anton Paar, Graz, Austria). NG samples were diluted in Milli-Q water (1:100) to avoid multiple scattering effects and measured in UVette Low Volume cuvettes (Eppendorf AG, Hamburg, Germany) at 25 °C. At least three runs for each sample were recorded, with at least 50 accepted readings [30,31]. The intensity distribution was determined by fitting the correlation data using a multi-exponential function (CONTIN), which accounts for the presence of multiple particle size populations. In this type of analysis, the presence of more than one family of particle sizes was considered. The ζ-potential was measured using the same instrument, employing an Omega cuvette (Mat. No. 225288, Anton Paar GmbH, Graz, Austria) by applying a voltage of 200 V and recording the electrophoretic mobility of the particles at 25 °C. The resulting mobility values were converted to ζ-potential using the Henry equation [32].

#### 2.4.2. Stability Measurement of NG

To evaluate the stability of NG formulations (S1, S2, and S3) under physiological conditions, particle size, PDI, and ζ-potential were measured immediately after preparation and after 21 days of incubation at 37 °C in a temperature-controlled incubator. Samples were stored in closed tubes without agitation throughout the incubation period.

#### 2.4.3. Scanning Electron Microscopy (SEM) Analysis

Aliquots of the samples were dried on glass slides under a controlled flow of inert nitrogen gas. The dried samples were then sputter-coated with a thin layer of gold. SEM imaging was performed using a Carl Zeiss NTS SUPRA 40 equipment (Oberkochen, Baden–Württemberg, Germany) operating at an accelerating voltage of 3 kV. Ultrastructural features of the particles were analysed at a magnification of 300×.

#### 2.4.4. Transmission Electron Microscopy (TEM) Analysis

A droplet of each formulation was carefully deposited onto a carbon-coated copper grid, and excess fluid was gently removed with filter paper. Samples were subsequently air-dried and subjected to negative staining to enhance contrast. Imaging was carried out using a Zeiss 109 TEM (Carl Zeiss NTS GmbH, Oberkochen, Germany) equipped with a Gatan W10000 camera. Representative micrographs were acquired at magnifications of 30,000× and 85,000× [33].

#### 2.4.5. Rheological Analysis

The viscoelastic properties of NG suspensions described in Table 1 were evaluated using an S3R301 rotational rheometer Anton Paar (Graz, Steiermark, Austria) (equipped with a 50 mm diameter cone–plate geometry (CP50-1/TG). The distance between the cone and the plate (gap) was set at 0.099 mm, which determined the sample volume analysed. The temperature was kept constant at 25 °C by a thermostatic control system.

The mechanical characteristics, by the viscoelastic storage (G′) and loss module (G″) as a function of angular frequency (ω) were measured. The damping factor (tan δ = G″/G′; δ: phase angle) was calculated as a function of frequency, with the aim of quantifying the relationship between the NG elastic and viscous behaviour. This parameter allowed to characterise the internal resistance of the system to deformations applied at different time scales.

On the other set of experiments, the variation in the apparent viscosity as a function of the shear rate (γ) was determined. The rheological behaviour under flow was analysed by applying a shear stress gradient in the range of 0 to 300 s^−1^. This analysis allowed us to identify the shear-thinning phenomenon characteristic of these NG suspensions. The data obtained were fitted to the power-law according to Equation (3):

(3) τ=K γ n

where *τ* is the applied shear stress (Pa), *K* is the consistency coefficient, *γ* is the shear rate (s^−1^), and n is the flow index, which indicates the degree of shear-thinning.

#### 2.4.6. Fluorescence Spectra

To investigate the characteristic fluorescence emission of CS-GNP NG, a Thermo Spectronic AMINCO-Bowman Series 2 spectrophotometer (Madison, WI, USA) was used. NG samples were placed in quartz cuvettes with a 1 cm optical path length. An excitation scan was performed in the range of 300–550 nm, recording the emission between 350 and 700 nm. Particular attention was given to the red emission region (500–700 nm), which has been reported in the literature as indicative of covalent complex formation between CS and GNP [34].

All measurements were performed at 25 °C. Control samples consisting of both single CS and GNP solutions were measured separately under identical instrumental conditions.

#### 2.4.7. Small-Angle X-Ray Scattering (SAXS)

SAXS measurements were carried out using a XENOCS Xeuss 2.0 UHR HP200 SAXS-USAXS/WAXS instrument (Laboratorio de Cristalografía Aplicada, ITECA Institute, CONICET-UNSAM, San Martín, Argentina). It is equipped with a GeniX3D Cu Ultra Low Divergence microfocus source (Cu-Ka X-ray beam, average wavelength λ = 0.15419 nm). This instrument allows simultaneous studies at small and wide angles (SAXS and WAXS, respectively) and it also has the possibility of measurements at ultra-small angles (USAXS). In the present work, only SAXS measurements were performed, using a Dectris Pilatus3 R 200K-A hybrid pixel photon counting detector (Dectris AG/Dectris Ltd., Baden-Dättwil, Switzerland). The sample-to-detector distance was set at 1200 mm, resulting in a scattering vector (q) range of 0.085–3.50 nm^−1^. These q-values were calibrated using a silver behenate standard. These experiments were performed in transmission geometry, using a high-flux configuration (beam size of 800 μm × 800 μm). Data reduction was performed using Foxtrot v3.4.9 software, and data fitting was carried out using SasView 6.0.1 software.

#### 2.4.8. Swelling Test

Dried NGs (S2) were immersed in ultrapure Milli-Q water and incubated at ambient temperature (25 °C). At predefined time points over a 120 h period, samples were removed, gently blotted to remove excess surface water, and weighed. The initial dry mass (*Wd*) of each sample was 0.01 g. The percentage of equilibrium swelling ratio (% *ESR*) was calculated using the following Equation (4):

(4) $$\%\;ESR=\left(\;\frac{Ws-Wd}{Wd}\;\right)\times 100$$

where

*Ws* is the mass of the swollen NG at time *t*;

*Wd* is the initial dry mass (0.01 g).

### 2.5. NGs in Biological Systems

#### 2.5.1. Cell Culture Conditions

Murine fibroblasts L929 (ATCC^®^CRL-2648 TM) and human retinal pigment epithelial ARPE-19 (ATCC^®^CRL-2302 TM) were maintained in a 37 °C incubator with 5% CO_2_ and high relative humidity (95–98%). Culture conditions: ARPE-19 and L929 cell lines were maintained in DMEM with 10% FBS, 1% GlutaMAX™ (Thermo Fisher Scientific (Gibco™ brand, Brooklyn, NY, USA)), 100 U/mL penicillin, and 100 μg/mL streptomycin. Culture medium was replaced three times per week, and cells were sub-cultured when they reached 70–80% confluence.

#### 2.5.2. Cell Metabolic Activity Determination

To evaluate the cytotoxicity of the NG, the MTT assay was performed. L929 and ARPE-19 cells were seeded in 96-well plates at a density of 1 × 10^4^ cells/well and 1.5 × 10^4^ cells/well, respectively, and were allowed to adhere for 24 h. The MTT assay was carried out in accordance with ISO 10993-5 standards, in which L929 fibroblasts are routinely employed for biocompatibility testing of nanosystems [35]. For L929 cells, the culture medium was replaced with fresh medium containing varying concentrations (5–150 µg/mL) of NGs corresponding to Samples 1, 2, and 3. For ARPE-19 cells, the medium was replaced with fresh medium containing 30 µg/mL of NG S2. Then, L929 and ARPE-19 cells were incubated with MTT at final concentrations of 0.25 mg/mL and 0.5 mg/mL, respectively, for 60 min at 37 °C in a 5% CO_2_ atmosphere. Finally, absorbance was measured at λ = 570 nm with background subtraction at λ = 690 nm on a POLARstar Omega micro plate reader (BMG LABTECH, Ortenberg, Germany).

#### 2.5.3. Cellular Internalisation

ARPE-19 cells were cultured in 24-well plates with sterile coverslips at a density of 3 × 10^4^ cells/well and allowed to adhere for 24 h. After reaching 60–70% confluence, a suspension of NGs (S2) at 30 µg/mL was incubated with the cells for different time points (5 min, 30 min, 4 h, 6 h, and 24 h).

After each incubation time, cells were rinsed three times with PBS to remove excess non-internalised NGs. Cells were fixed with 4% paraformaldehyde/4% sucrose (PFA-S) in PBS for 20 m. Then, nuclei were counterstained with the DAPI fluorescent probe. Finally, the coverslips were washed with PBS and mounted with 2.4% mounting medium (mowiol). The uptake of NGs by ARPE-19 cells was studied using an Olympus IX71 microscope (Olympus Corp., Tokyo, Japan). For image analysis, Fiji (an ImageJ-based software, NIH, version 1.54f) was used to quantify the mean fluorescence intensity of NGs per cell by manually delineating cell boundaries. To account for differences in cell size, the fluorescence values were normalised using an area correction factor. Final intensity values were reported as the average per cell for each treatment. Image contrast and brightness were uniformly adjusted across all images using Adobe Photoshop 8.0.1. (San José, CA, USA: Adobe Systems Incorporated).

### 2.6. Statistical Analysis

All experiments were performed in triplicate (*n* = 3), unless otherwise specified. Statistical analyses were conducted using GraphPad Prism version 8.3.0 (San Diego, CA, USA). Data were subjected to analysis of variance (ANOVA) and the Kruskal–Wallis test. Post hoc comparisons were carried out using either the Mann–Whitney test or Tukey’s test, with significance set at *p* ≤ 0.05.

## 3. Results and Discussion

### 3.1. NG Construction and Kinetics of Formation

**Figure 1:**
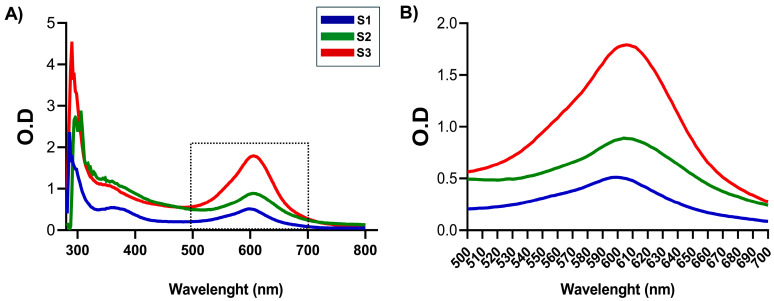
(**A**) Visible spectra for CS-GNP corresponding to S1, S2, and S3 samples. (**B**) Visible zone zoom of spectra, range of 500 nm–750 nm showing the peak between 590 and 620 nm.

Firstly, visible spectra were obtained to identify a specific absorbance peak at near 600 nm [14,25,36] for each CS-GNP NG sample (Figure 1A). This phenomenon was linked to the formation of Schiff bases, responsible for the final stability of the polymer network after GNP reacted with -NH2 groups of CS. Among the samples examined, S1, S2, and S3 showed the characteristic maximum at λ ≅ 600 nm (Figure 1B, Supplementary Figure S1), indicating that NG formulation was adequate as linked GNP absorbs at this wavelength [37].

**Figure 2:**
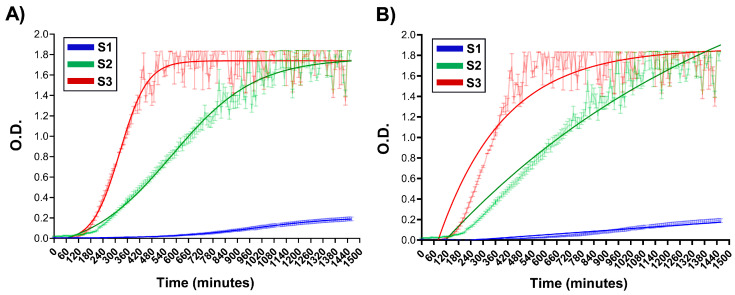
Optical density (O.D.) versus time curves for selected formulations, chosen based on kinetic behaviours observed visually (**A**) Sigmoidal fitting application. (**B**) First-order fitting application. Shaded areas represent experimental data, while solid-coloured lines correspond to the fitted curves.

In other set of experiments, the variation in O.D. at λ = 600 nm was registered during 24 h. As an example for the reader, the O.D. vs. time curves for samples S1, S2, S3 are shown (Figure 2) since they present different kinetic behaviours, which was evident with simple observation.

Kinetic models were applied to the experimental data to determine parameters describing NG formation: Sigmoidal Kinetics (SK) (Figure 2A) and First-Order Exponential (FOE), (Figure 2B) [16,28]. All kinetic parameters obtained by applying the specified models are summarised in Table 2. First of all, it is worth noting that both SK and FOE exhibited high R^2^ values. However, SK presented a better correlation for the exponential phase, capturing the transition more accurately. The FOE model does not appear to fit correctly to the initial phase, which is particularly notorious for S2 and S3. The first-order fitting underestimates the initial rate, which coincides with the results obtained in the rate comparison. In the *plateau* phase, both models converge and predict similar values at maximum absorbance. Therefore, we consider that the formation kinetics of CS-GNP NG have sigmoidal behaviour with 3 well-defined phases. With the presence of a lag phase followed by accelerated growth and then stabilisation, the four-parameter sigmoidal equation is deemed the most appropriate model to describe the formation kinetics.

As summarised in Table 2, sample S1 exhibited the lowest NG formation rate determined as the slope (O.D./min, with units of t^−1^), whereas S3 displayed the highest. These findings support the role of the CS’s pH as a key determinant in the kinetics of NG formation, in alignment with recent reports on the progression of CS-GNP crosslinking [16].

### 3.2. Chemical Groups Interaction

**Figure 3:**
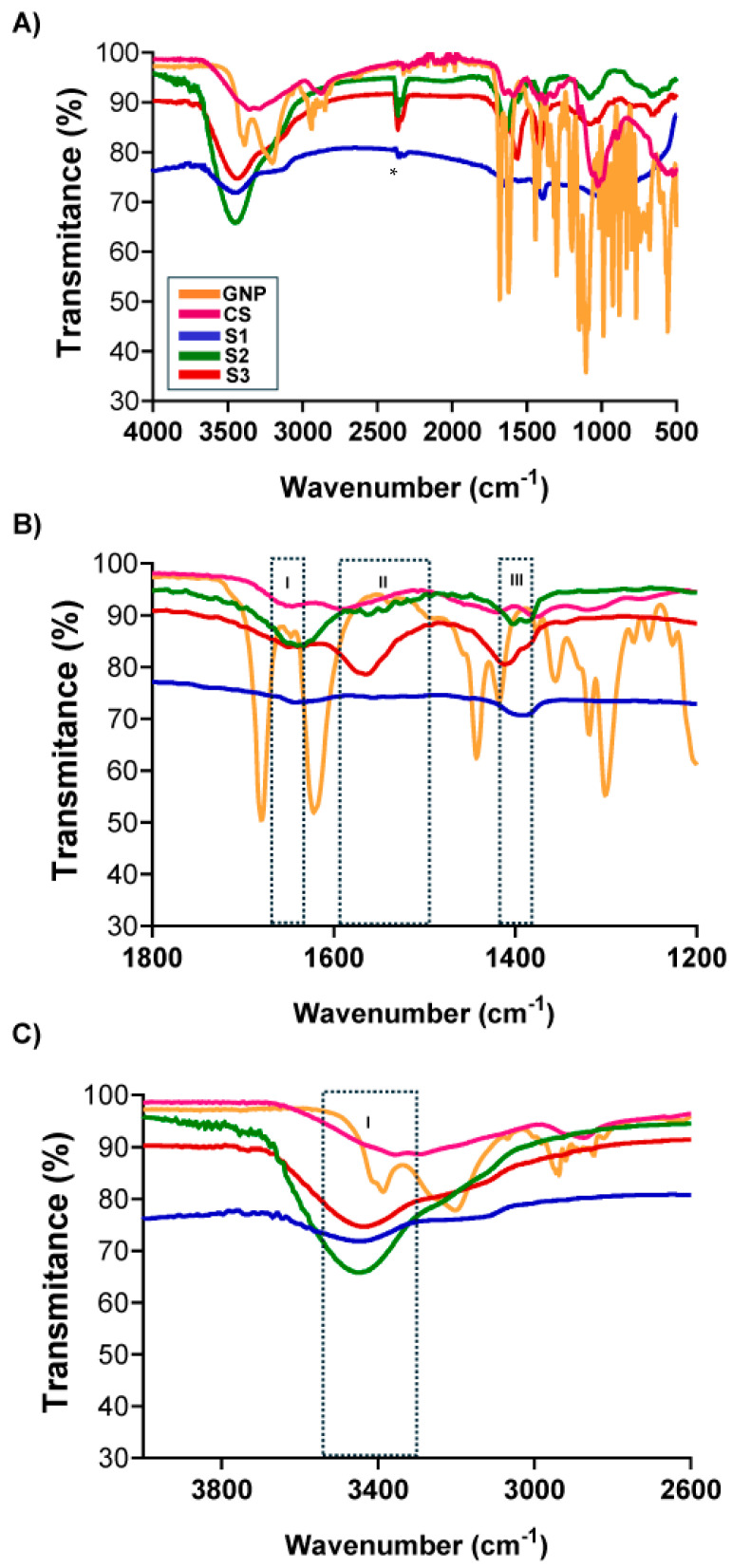
FTIR-ATR spectra of CS, GNP, as well as S1, S2, and S3. (**A**) Full spectra in the range of 4000–500 cm^−1^. (**B**) Zoomed region between 1800 and 1200 cm^−1^ highlighting amide (I–III) bands associated with crosslinking interactions. (**C**) Enlarged view of the 3800–2600 cm^−1^ region, showing N-H and O-H stretching vibrations and their variation across the formulations. The asterisk (*) indicates signals corresponding to atmospheric carbon dioxide.

The chemical groups involved in the chemical interactions contributing to NG formation were investigated through the analysis of their FTIR spectra. Figure 3A shows the comparative FTIR-ATR spectra of GNP, CS, and NG formulations for S1, S2, and S3. For single CS and GNP, the characteristic bands previously reported were identified [29]. The chemical groups involved in the chemical interactions contributing to NG formation were investigated through the analysis of their FTIR spectra. Figure 3A shows the comparative FTIR-ATR spectra of GNP, CS, and NG formulations for S1, S2, and S3. For single CS and GNP, the characteristic bands previously reported were identified [38]. When compared with NG, notable changes were observed in the regions associated with amino (-NH_2_)-, hydroxyl (-OH)-, and amide (-CONH_2_)-type bonds, indicating the formation of covalent bonds between CS and GNP. In the region of 3500–3200 cm^−1^ (Figure 3C, box I), mainly attributed to N-H and O-H stretching, a marked decrease in intensity was recorded for all NGs compared to CS. This change reflects the consumption of -NH_2_ and -OH groups due to their reaction with GNP, and possibly the formation of internal hydrogen bonds within the crosslinked network [38,39]. The intensity reduction was more pronounced in S1 and S3, suggesting a different interaction dynamic under varying pH conditions. In the range of 1800–1200 cm^−1^ (Figure 3B, boxes I–III), characteristic vibrational changes further confirm the crosslinking process. In box I (~1650–1600 cm^−1^), the C=O stretching band (Amide I) shifted towards ~1640 cm^−1^ and exhibited increased intensity in S1, S2, and S3 compared to CS, consistent with the formation of new amide bonds [39]. Box II (~1560 cm^−1^) revealed more defined bands in all NGs, attributed to Amide II and/or Schiff base formation (C=N), suggesting enhanced reaction with primary amino groups [39]. Box III (~1420–1370 cm^−1^) displayed variations in CH_2_/CH_3_ deformation and polymer ring vibrations, indicating structural rearrangements in the polymer matrix and crosslinking degree [40].

Together, these shifts and new signals corroborate that GNP reacts covalently with CS to form amide and Schiff base-type structures [38,41]. The reduced intensity in the N-H/O-H region further supports the conversion of reactive groups into crosslinking sites. While all three formulations show spectral features consistent with successful crosslinking, slight differences in peak intensity and position among S1, S2, and S3 may reflect the influence of pH on reaction kinetics and network formation. These findings align with previous studies on CS-GNP hydrogels [29,38] and confirm the successful generation of NGs under varying formulation conditions.

### 3.3. Particle Size Distribution, PDI and ζ-Potential

DLS measures the time-dependent fluctuations in the intensity of scattered light resulting from Brownian motion of particles in suspension. Consequently, the fundamental information obtained from DLS is based on the intensity of scattered light. Since the scattering intensity is proportional to the sixth power of the particle diameter, larger particles (even in small amounts) contribute disproportionately to the overall signal. While DLS software can mathematically transform intensity data into volume or number distributions, these conversions tend to overestimate peak widths, and the resulting values are less reliable. Therefore, it is recommended to use intensity-based size distributions when reporting the size of each mode, especially in polydisperse systems [42].

**Figure 4:**
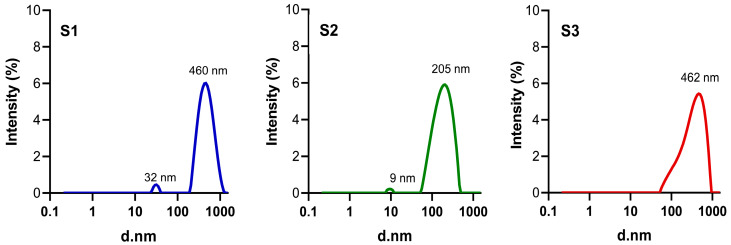
Particle size distribution in terms of intensity versus diameter (d.nm) of CS-GNP for S1, S2, and S3 samples, measured by DLS. The main peak values for each formulation are indicated within the plots, highlighting the differences in average hydrodynamic diameter and aggregation behaviour among the samples. DLS measurements were performed at room temperature (~25 °C) using ultrapure water at the corresponding pH value.

Based on intensity measurements, the obtained size distribution profiles revealed distinct behaviours among the three formulations (Figure 4). S1 and S3 exhibited relatively narrow distributions with dominant peaks at 460 nm and 462 nm, respectively, and minor populations at 32 nm (S1) and 9 nm (S2), indicating the presence of some small nanostructures. S2 displayed a more defined bimodal profile, with peaks at 9 nm and 205 nm, suggesting a better dispersion profile compared to the other two formulations. Remarkably, HIUS treatment results highlight its essential role in obtaining structures falling into nanoscale, i.e., disintegration of NG clusters. The absence of this step implies formulations with sizes > 1000 nm and multimodal distributions (Supplementary Figure S2).

The polydispersity index (PDI) represents a key parameter in the design and synthesis of particles. This dimensionless value ranges from 0, reflecting a highly homogeneous size distribution, to 1, indicating significant size variability within the formulation [43]. Regarding suspension stability, formulations are generally considered stable when the absolute value of the ζ-potential exceeds ±25 mV [40]. Table 3 shows the PDI and ζ-Potential of NGs generated from the different CS and GNP combinations studied. Formulation S2 presented a more favourable profile, with an acceptable PDI (0.252 ± 0.024) and a ζ-potential of 29.35 ± 1.17 mV, a significantly smaller hydrodynamic size as shown before, suggesting adequate dispersion and stability. Sample S3 exhibited behaviours with specific limitations in terms of size that determined their exclusion for further uses. S3 had a hydrodynamic diameter of 304.90 ± 27.44 nm and a moderate PDI of 0.274 ± 0.033. Its ζ-potential was 20.42 ± 0.75 mV, which falls below the ±25 mV threshold generally considered necessary to ensure adequate colloidal stability, which could increase the risk of aggregation over time [44]. Although sample S1 exhibited a ζ-potential above the stability threshold (31.64 ± 1.43 mV), it also showed a minor population of larger particles approaching the upper nanoscale limit, along with a PDI of 0.229 ± 0.035. While its PDI was acceptable, the broad size distribution and the proximity to the microscale range reduced its overall suitability for NG applications where size uniformity and compact dimensions are critical [45,46].

### 3.4. Stability of NG Formulation Under Physiological Temperature Conditions

To evaluate the colloidal stability of NG over time, samples were incubated at 37 °C for 21 days, and changes in particle size distribution and ζ-potential were assessed (Supplementary Figure S3).

Supplementary Figure S3A shows the visible spectra of S1, S2, and S3 after 21 days of incubation at 37 °C. All samples exhibited low overall absorbance (O.D. < 0.3) across the measured range, with no sharp indicative peaks of molecular chromophores. However, a broad and shallow increase in signal was observed around 680 nm, which may reflect enhanced light scattering that could be associated with a possible particle aggregation or increased turbidity.

Supplementary Figure S3B reveals that S1 and S3 underwent a marked shift towards larger and more polydisperse populations. S1 exhibited a major peak at 1034 nm along with additional smaller peaks at 13 and 58 nm, while S3 displayed a dominant peak at 747 nm and a minor population at 47 nm. These changes are indicative of particle aggregation or fusion over time. In contrast, S2 maintained a narrower distribution, with a main peak at 586 nm and a minor population at 32 nm, reflecting a comparatively lower increase in size relative to S1 and S3.

ζ-potential analysis further supported these findings. Notably, S2 showed a statistically significant increase in surface charge over time (from ~30 to ~38 mV, *p* < 0.01), suggesting improved electrostatic stabilisation (Supplementary Figure S3C). This increase in ζ-potential may be attributed to structural rearrangements at the NG surface, potentially exposing more protonated –NH_2_ groups of chitosan. This shift toward a higher surface charge could contribute to enhanced colloidal stability by strengthening electrostatic repulsion between particles. (Supplementary Figure S3C). The observed increase in ζ-potential in S2 over 21 days may be attributed to structural rearrangements at the NG surface, potentially exposing more protonated -NH_2_ groups of CS. This shift toward higher surface charge could contribute to enhanced colloidal stability by strengthening the electrostatic repulsion between particles [47]. Overall, S2 seems to exhibit acceptable colloidal stability under physiological temperature conditions, while S1 and S3 experienced size enlargement and broader distributions, consistent with aggregation phenomena.

### 3.5. Ultrastructural Analysis of NG

**Figure 5:**
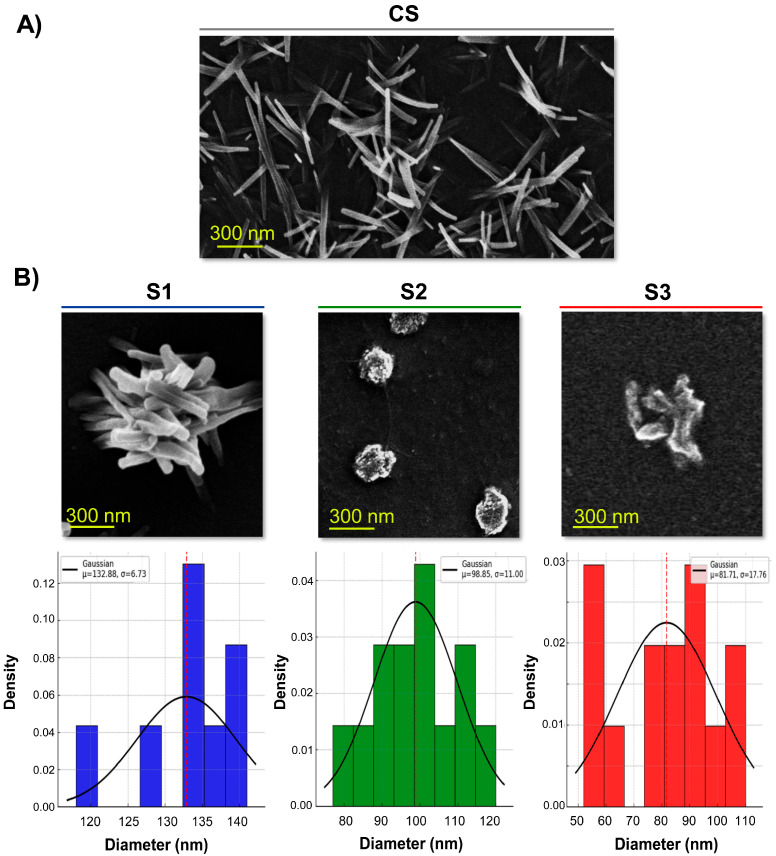
(**A**) Representative SEM images of single CS and NG (S1, S2, and S3). (**B**) Histograms representing particle size distributions for the three NS samples, each overlaid with a fitted Gaussian curve. Since the histograms were normalised, the Y-axis was labelled as density, and the bars were scaled so that the total area under each histogram equals 1. Red-dashed red lines indicate the mean particle size for each distribution.

The micrographs obtained by SEM (Figure 5) show that all NG samples generated at different pH values (S1, S2, and S3) exhibited dimensions predominantly within the nanoscale range, although notable morphological differences can be observed. The non-crosslinked CS, single-polysaccharide samples displayed elongated and fibrous structures arranged in an apparently random manner (Figure 5A). The surface appeared rough and irregular, likely due to the disorganised arrangement of CS chains in the absence of GNP-mediated crosslinking coinciding with the observations reported by [28]. These observations support the notion that single CS, without the addition of GNP, did not form structured NGs, but instead retained a more fibrillar or entangled morphology prone to aggregation. In S1, a denser aggregation of fibrous structures and irregular shapes was observed, suggesting enhanced interactions between polymer chains, while no spheroid structures were evident. In S2, well-defined nanometric particles were obtained through the image analysis of 111.02 ± 11.26 determining a globular to slightly oval morphology. These structures appeared as individual and compact units, with smooth contours and slight surface irregularities, suggesting the crosslinking process between CS and GNP. No evidence of faceted shapes was detected, reinforcing the idea of controlled molecular packing favoured by the optimised pH conditions. In contrast, S3 exhibited a markedly different morphology, characterised by smaller, elongated, and sparsely distributed nanostructures. The reduced size (estimated in 104.67 ± 20.31 nm from the image) and lower density of particles suggest a less efficient crosslinking process, resulting in a more fragmented and less cohesive network (Figure 5B). The SEM analysis indicates that S2 would be the most homogeneous formulation in terms of size, whereas the pH of S3 shows a more fragmented and dispersed architecture, which could compromise its colloidal stability.

**Figure 6:**
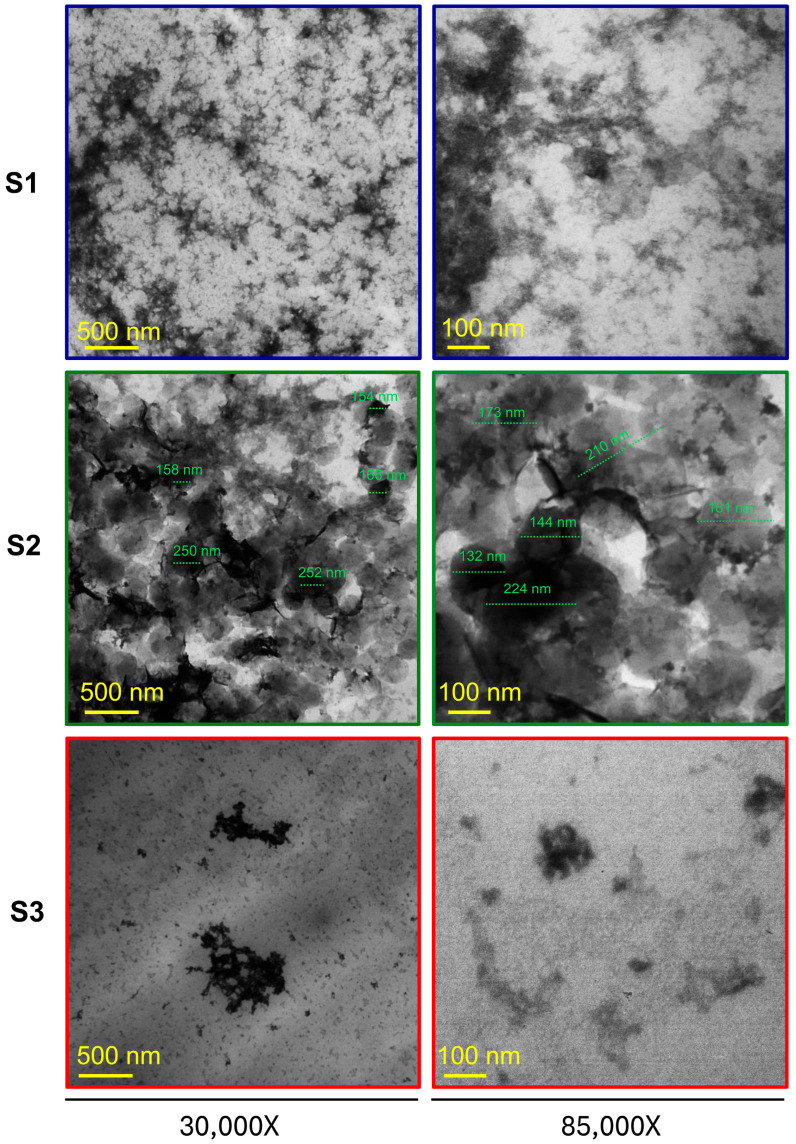
TEM images of NG samples S1, S2, and S3 at 30,000× and 85,000× magnification. Scale bars are included in the images. For S2, individual nanogels were measured directly from the micrographs to estimate particle size.

To corroborate the morphologies of samples S1, S2, and S3 using an alternative method, a TEM analysis was conducted. As shown in Figure 6, images provided complementary ultrastructural insights at higher resolution, confirming the nanoscale dimensions and characteristic shapes of the NG observed by SEM. For S1, the TEM micrographs revealed irregular fibrillar structures without defined borders. In S2, individual NGs appeared as well-delineated spherical or slightly oval particles, with a dense core and smooth outline. This morphology aligns with the hypothesis of effective ionic crosslinking between CS and GNP under optimised pH conditions. In the case of S3, the particles were less uniform, often exhibiting elongated shapes with variable contrast and spacing, indicative of partial structural organisation and a less compact network. Altogether, the TEM analysis supports the morphological trends identified by SEM, reinforcing the notion that formulation conditions significantly influence NG shape and homogeneity.

In sum, both SEM and TEM analyses highlight the critical influence of pH on the efficiency of NG formation and the resulting morphology. The observed morphological variations underscore the role of pH in modulating the structure and stability of NGs by influencing both the degree of crosslinking and the final particle organisation. The differences among S1, S2, and S3 can be attributed to the pH-dependent protonation state of CS, which governs the electrostatic environment and the efficiency of crosslinking with GNP. This is supported by Miras et al. (2021) [23], who demonstrated that CS-GNP nanofilms exhibit significant pH-responsive structural and mechanical changes. Furthermore, Delmar and Bianco-Peled (2015) [16] showed that even small variations in pH (from 4.00 to 5.50) dramatically affect the gelation kinetics and equilibrium swelling of CS-GNP hydrogels, with a 1.5-unit increase in pH leading to nearly a fourfold decrease in gelation time and more than a tenfold increase in swelling capacity. These findings reinforce the notion that subtle shifts in formulation pH can lead to distinct morphologies, even when the polymer and crosslinker concentrations are kept constant. Although neither study provides direct morphological imaging (e.g., SEM or TEM), their data on pH-dependent physicochemical properties strongly suggest underlying structural rearrangements, which are likely reflected in the morphological differences observed in our SEM analysis.

SEM and TEM analyses, jointly with its ζ-potential and size obtained by DLS, supports the criterion of selecting the S2 as the most appropriate formulation [16]. An important consideration is that the drying process required for SEM and TEM sample preparation leads to “NG dehydration,” causing NG to shrink, displaying reduced sizes compared to their hydrated state [48]. In contrast, DLS measures the hydrodynamic diameter of NG in an aqueous environment, where they maintain their fully hydrated structure. This slight difference in sample state explains the size discrepancies observed between SEM/TEM and DLS measurements [49].

### 3.6. NG Rheological and SAXS Insights

**Figure 7:**
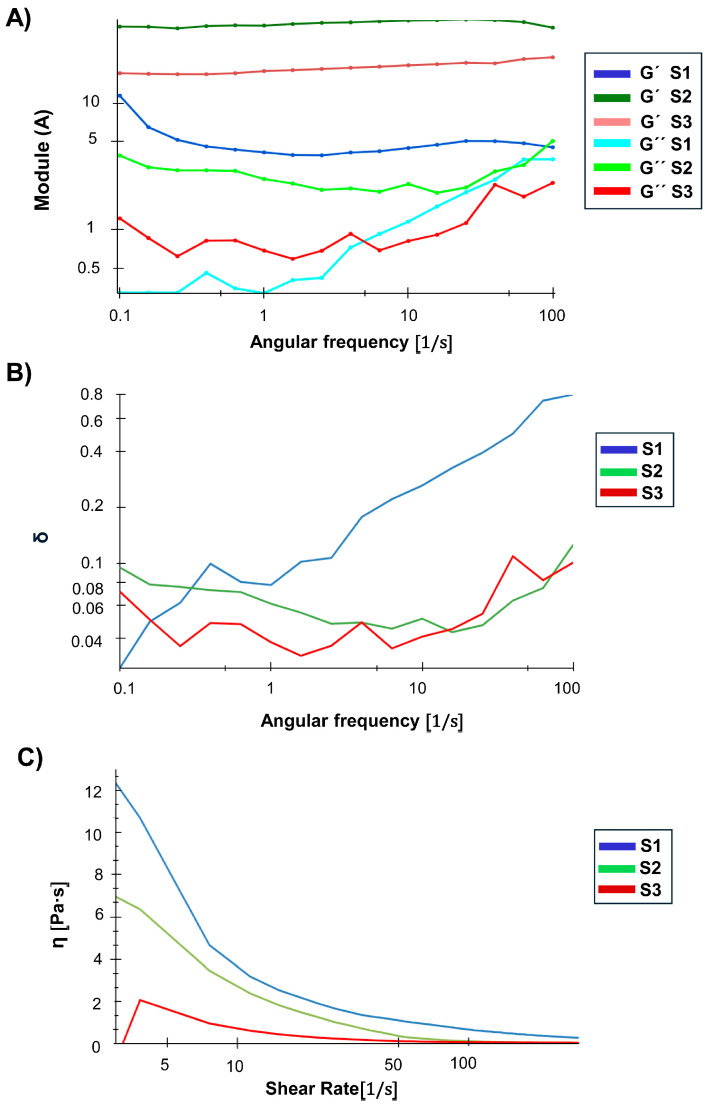
(**A**) Frequency sweeps of the NG (S1, S2, S3) suspensions, showing the evolution of G′ (elastic modulus) and G″ (viscous modulus) as a function of angular frequency. (**B**) Plot of tan δ (G″/G′) vs. angular frequency. (**C**) Flow curves of the NG suspensions, showing the apparent viscosity (η) as a function of shear rate (0–300 s^−1^).

Rheological assays are fundamental to evaluating the mechanical stability and viscoelastic behaviour of NG suspensions under dynamic conditions. In this context, the storage modulus (G′) represents the elastic response, the loss modulus (G″) reflects the viscous behaviour, and the damping factor (tan δ = G″/G′) indicates the balance between solidity and fluidity of the system [50]. The frequency sweeps (Figure 7A) evidenced remarkable differences in the mechanical response of the NGs analysed in S1, S2, and S3. S2 presented the highest G′ values over the entire frequency range, with a lower frequency dependence in comparison with the remaining formulations, reflecting the establishment of a dense and stable polymeric network suspended in the liquid bosom, with G′ greater than G″ over the entire frequency interval studied. This behaviour suggests that S2 would be the most resistant system to mechanical deformations, maintaining its structure without significant transitions towards a more fluid state. In S3, a lower solid character was observed compared to S2, but with G′ still predominant over G″ over most of the frequency interval. However, at higher frequencies, G′ and G″ come closer, indicating a partial loss of the crosslinked structure under dynamic mechanical stress. This suggests that S3 would be more susceptible to fluidity under high deformation conditions. In turn, S1 showed lower G′ than S2 and S3, with a higher frequency dependence and a significant reduction in this parameter in the high frequency domain. S1 showed a tendency to lose its elastic character as the frequency increases, indicating that its polymeric network is more susceptible to mechanical deformation. Although G′ still predominates over G″ throughout most of the range, at high frequencies, G″ approaches G′, which could favour a more fluid behaviour under high mechanical stress stimuli. To complete the comparison, G′ value was evaluated at a fixed frequency value of 10 Hz (Table 1). It was seen that the solid character of the S1 suspension exhibited ten percent of the value detected for S2. Meanwhile, S3 manifested an intermediate value.

The damping factor (tan δ) or relative viscoelasticity, defined as the ratio between the viscous modulus (G″) and the elastic modulus (G′), provides key information about the viscoelastic nature of the samples (Figure 7B). S1 showed a progressive increase in tan δ as the frequency increases, indicating a gradual transition towards a more fluid behaviour under this condition of deformation, i.e., at high frequencies its structural network weakens and allows for greater fluidity. S2 presented the lowest tan δ values across the frequency range, confirming its predominantly solid elastic behaviour and higher resistance to mechanical deformation. S3 showed intermediate tan δ values at low frequencies, but with fluctuations and a progressive increase at high frequencies, indicating a less stable polymeric network upon frequency deformation with a greater propensity to lose stiffness under conditions of prolonged mechanical stress. Taken together, these rheological analyses indicate that S2, generated at pH 4.5, would be the system with the highest mechanical stability and lowest frequency dependence, making it more suitable for applications requiring a structure resistant to prolonged mechanical stress. However, S1, with its high viscosity and tendency to exhibit fluidity under certain conditions, might be preferable in applications requiring higher initial flow resistance. Finally, S3, with its lower structural stability and greater tendency to flow, would be more suitable for applications where deformability and low viscosity are key factors [46,51,52].

The flow curves (Figure 7C) provided additional information on the dependence of viscosity on shear rate. All samples exhibit pseudoplastic behaviour, characterised by the decrease in viscosity with increasing shear rate, which was pointed out as typical for polymeric systems and NGs [53,54]. S1 presented the highest viscosity, indicating a higher initial resistance to flow and a dense structure. However, this result does not imply greater mechanical resistance to deformation, as frequency sweeps showed that S1 has a network more dependent on mechanical stress, which could make it more susceptible to deformation under certain conditions. On the other hand, S2 presented an intermediate viscosity, lower than that of S1 but higher than that of S3. Its lower viscosity compared to S1 suggests that its polymeric network has a better structural organisation, allowing it to maintain a stable structure without requiring extreme viscosity as a result of the deformation applied. Finally, S3 showed the lowest viscosity, confirming that it is the most fluid system with the lowest degree of crosslinking between the NG entities. Taken at the final asymptotic portion of the curve, the highest *η* was registered for S1, while S2 and S3 exhibited very similar results (Table 1).

**Figure 8:**
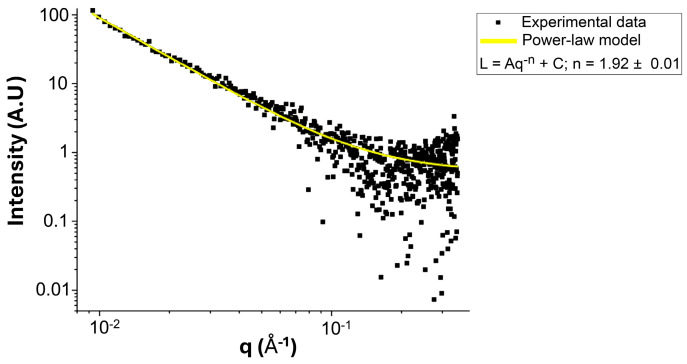
SAXS profile of NG S2, showing experimental data (black squares) and the corresponding power-law fitting (yellow line). The fitted curve follows the equation I = Aq^−^ⁿ + C, with an exponent n = 1.92 ± 0.01, suggesting a mass-fractal structure in the analysed range.

Figure 8 shows SAXS data observed for the NG sample S2, while no signal was observed after a few hours. The obtained SAXS curve was fitted using a power-law model, commonly employed for the analysis of disordered polymeric networks and nanogels [55]. Using this model, an exponent n = 1.92 ± 0.01 was obtained, which is consistent with scattering from a soft, disordered polymer network with mass-fractal characteristics [55]. The value is in line with mass-fractal exponents reported for polymer gels based on fractal models developed by Beaucage [56].

### 3.7. Swelling Behaviour of NGs

Characterising the swelling behaviour of NGs provides critical insights into their structural dynamics, hydration capacity, and suitability for sustained drug delivery, as swelling influences both release kinetics and interaction with biological environments [57]. Particularly, Butler et al. (2006) [58] exposed that the swelling behaviour of CS-based NG is strongly pH-dependent due to the polymer’s polyelectrolytic nature. Below the pKa (~6.3), protonated -NH_2_ groups generate positive charges that drive water uptake via osmotic forces, with maximum swelling near pH 3. Above pH 6.5, deprotonation reduces charge density, and swelling becomes negligible.

In this study, the swelling behaviour was monitored on S2 formulation over 120 h by measuring its hydrated mass under aqueous conditions. As summarised in Table 4, a progressive increase in mass was observed during the initial 24 h, with the equilibrium swelling ratio (ESR) reaching 2933.3%. This value increased slightly thereafter, plateauing at approximately 3100% between 72 and 120 h. Also, from 48 to 72 h, the swelling index clearly reaches a *plateau*, indicating that the NGs achieved their maximum hydration capacity. This equilibrium behaviour agrees with reports by Liu et al. (2014) [59] and Chen et al. (2013) [60], who observed a complete swelling of CS-GNP networks within 24–72 h under aqueous conditions.

### 3.8. Biocompatibility Assays

#### 3.8.1. Impact of CS-GNP NG on Cell Viability

To determine the biocompatibility of the developed NG for potential uses in food and/or pharmaceutical applications, cytotoxicity assays were conducted in accordance with ISO 10993 guidelines [35], which provide a standardised framework for the in vitro evaluation of medical device materials and substances. Specifically, the ISO 10993-5:2009 standard recommends the use of the murine fibroblast cell line L929 as a reference model for assessing cytotoxic potential. In compliance with these recommendations, L929 cells were exposed to a range of nanoparticle (NP) concentrations under standardised culture conditions to evaluate cell viability, proliferation, and morphological integrity. The concentration-dependent effects were quantified using established viability assays, such as MTT or Alamar Blue, and cytotoxicity thresholds were defined based on the reduction in cell viability relative to untreated controls. A material is considered non-cytotoxic according to ISO criteria when cell viability remains above 75% following exposure [35].

L929 cells are characterised by a rapid growth, producing confluent monolayers. The adherent cells displayed a spindle shape and epithelial-like morphology. Common morphological changes associated with cellular stress and/or toxicity include an initial rounding followed by cell detachment, as well as decreased confluence [61]. Fortunately, none of these alterations were observed following CS-GNP NG treatment at concentrations ≤ 100 µg/mL.

**Figure 9:**
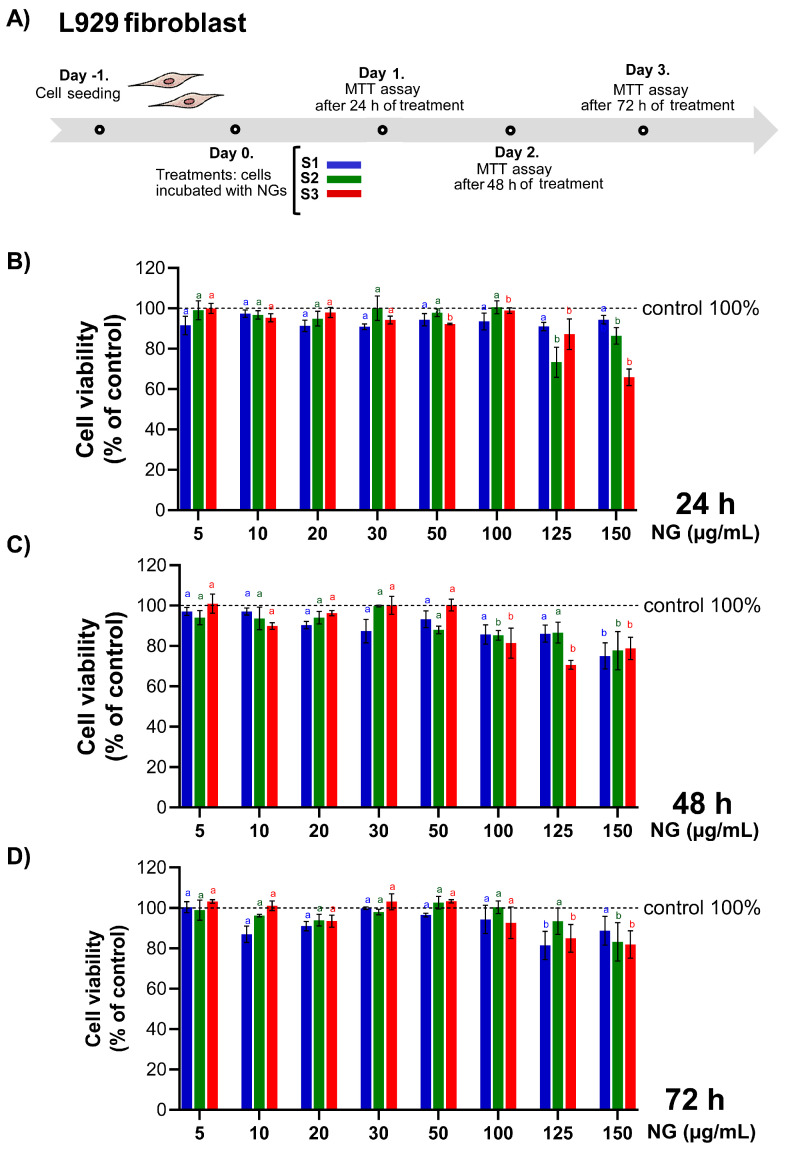
Assessment of cell viability upon exposure to nanogels (NGs). (**A**) Experimental timeline for MTT assay performed on L929 dermal fibroblasts following incubation with nanogel formulations S1, S2, and S3. Cells were seeded on Day 1 and treated on Day 0. MTT assays were conducted after 24, 48, and 72 h of treatment. Cell viability (%) after (**B**) 24 h, (**C**) 48 h, and (**D**) 72 h of exposure to increasing concentrations (5–150 µg/mL) of NG formulations. Viability is expressed as percentage relative to untreated control (100%, dashed line). Different letters indicate statistically significant differences between concentrations within each formulation group (*p* < 0.05); identical letters denote no significant difference. Letters are colour-coded to match each sample (S1: blue, S2: green, S3: red).

MTT assays were conducted on L929 dermal fibroblasts exposed to nanogel formulations (S1, S2, and S3) at concentrations ranging from 5 to 150 µg/mL for 24 h, 48 h, and 72 h (Figure 9A–C). Across all formulations, concentrations between 5 and 100 µg/mL did not induce significant reductions in cell viability compared to the untreated control (set at 100%), with mean values consistently above 90%, indicating good cytocompatibility. However, a concentration-dependent decline in viability was observed from 125 µg/mL onwards, with values falling below the threshold defined by ISO 10993-5 as indicative of cytotoxicity. This effect was most evident at 24 h and 48 h, suggesting that the cytotoxic response occurs rapidly upon exposure to high NG concentrations. Notably, viability values at 72 h did not show a further decrease and, in some cases, appeared slightly higher than those at earlier time points. However, this apparent plateau must be interpreted with caution due to the increased variability observed at 72 h, as evidenced by the broader error bars. This variability may reflect the combined influence of contact inhibition, nutrient depletion, and increased heterogeneity in NG–cell interactions over time in confluent cultures. These factors could impact mitochondrial metabolic activity and contribute to intra-assay differences in the MTT signal. Although CS and GNP are natural and generally recognised as biocompatible, the observed decrease in cell viability is influenced by multiple factors such as particle size, surface charge, morphology, and dose (Del Prado-Audelo et al., 2019) [62], underscoring the need for careful optimisation of formulation parameters depending on the target application and cell model. In this context, we report that cellular responses to free CS or CS-based nanosystems vary notably across cell lines, with some exhibiting proliferative effects (e.g., Caco-2, EA.hy926, hRPE-1, ARPE-19) [20,63], while others, such as SH-SY5Y neuroblastoma cells, show mild cytotoxicity 100 µg/mL with viability values at about 85% [21,64]. In L929 fibroblasts, similar trends have been observed, where certain nanocomposite formulations induced the reduction of cell viability over time, particularly at higher NP loading [65].

#### 3.8.2. Cellular Internalisation

**Figure 10:**
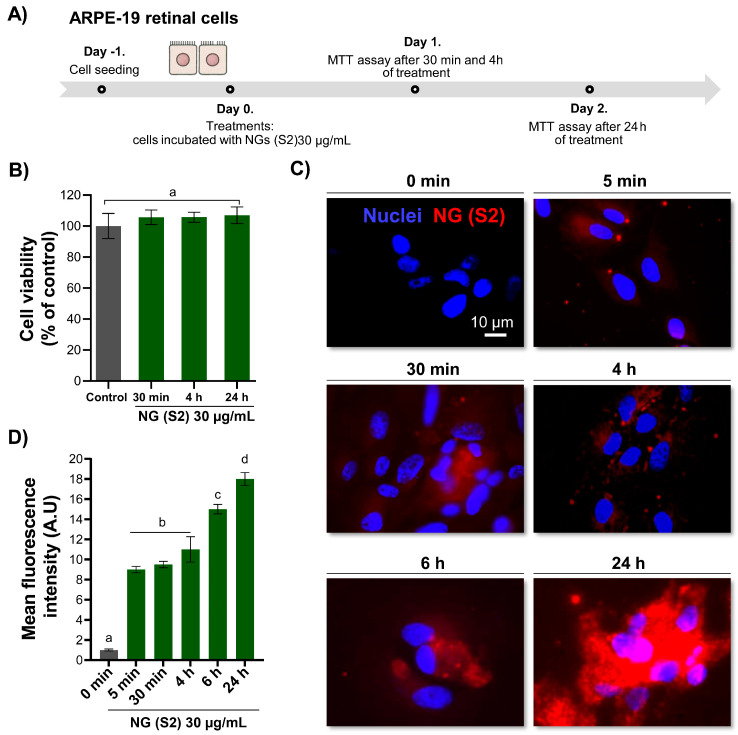
Cellular uptake and metabolic activity of ARPE-19 cells exposed to 30 µg/mL NGs (S2). (**A**) Schematic timeline of the experimental design. ARPE-19 cells were seeded and incubated with NGs at different time points. (**B**) MTT assay performed at 30 min, 4 h, and 24 h after exposure of L929 fibroblasts to NG formulations, showing no cytotoxicity at any time point. (**C**) Representative epifluorescence microscopy images showing gradual cellular uptake in NGs (red) in ARPE-19 cells at 5 min, 30 min, 4 h, 6 h, and 24 h. Nuclei were stained with DAPI (blue). Scale bar: 10 µm. (**D**) Quantification of cellular uptake represented as mean fluorescence intensity over time. Different letters indicate statistically significant differences between time points (*p* < 0.05).

In nanomedicine, the safe and efficient cellular internalisation of nanoparticles (NPs) is a critical step toward achieving high therapeutic efficacy when used as drug delivery systems [66]. In this study, the uptake capacity of NG S2 (30 μg/mL) was evaluated over time (0, 5 min, 30 min, 4 h, 6 h, and 24 h) in ARPE-19 cells. As outlined in the schematic representation of the experimental design (Figure 9A), treatments were applied under identical conditions, and MTT assays confirmed the absence of cytotoxic effects at all time points (Figure 10B). Representative epifluorescence microscopy images shown that NGs were gradually internalised over time, with widespread uptake evident after 24 h of incubation (Figure 10C). Fluorescence intensity, used as a proxy for NG uptake, showed a progressive increase that peaked at the final time point (Figure 9D). These findings are in line with previous results obtained by our research group using the same cell line (ARPE-19) in studies involving a different encapsulation platform, namely CS-based NGs crosslinked with tripolyphosphate (TPP) [20]. In that report, the internalisation of the nanosystems was also confirmed, suggesting consistent cellular uptake behaviour across structurally distinct yet functionally related nanocarrier systems. The agreement between the two independent approaches not only reinforces the reliability of the experimental methodology employed but also highlights the reproducibility and robustness of the internalisation process in this retinal epithelial cell model. Taken together, these results suggest that the cellular uptake observed is largely governed by the intrinsic properties of the CS-based NGs and their physicochemical compatibility with the target cell type, rather than being exclusively dependent on the specific crosslinker used.

## 4. Conclusions

This study enabled the comprehensive design, generation, and characterisation of CS-GNP NG, optimising critical parameters such as particle size, ζ-potential, colloidal stability, swelling behaviour, and rheological properties. While the covalent crosslinking of CS with GNP was previously described, the present work introduces a simplified and reproducible protocol performed entirely at 37 °C after 24 h, representing a substantial improvement over conventional approaches that require elevated temperatures and prolonged reaction times. The mild reaction conditions not only enhance biomedical compatibility but may also contribute to minimising aggregation and preserving NG integrity.

Among the key findings, the CS solution’s pH was identified as a crucial determinant of crosslinking kinetics and final network structure. In particular, pH 4.5 (S2 formulation) led to the formation of compact, homogeneous NGs with favourable physicochemical characteristics. Structural analyses (FTIR, SAXS, SEM, and TEM) confirmed the formation of covalent crosslinks, specifically Schiff base and amide bonds, defined spherical morphology and a compact internal organisation. The swelling study further supported these findings, revealing that formulation S2 achieved a stable hydration state within a relatively short period. Notably, rheological characterisation revealed a pseudoplastic behaviour, which is highly desirable for biomedical formulations. This property facilitates the application and spreading of the NG on biological tissues, enhances injectability, and improves patient compliance in topical or mucosal delivery systems. Furthermore, such flow behaviour is advantageous during industrial processing, ensuring ease of pumping, dosing, and filling under variable shear conditions without compromising formulation consistency [67]. Additionally, biocompatibility assays indicated low cytotoxicity of up to 100 µg/mL, whilst cellular uptake studies demonstrated efficient, time-dependent internalisation, thereby further supporting their potential for intracellular drug delivery.

Altogether, the optimised CS-GNP NG combines structural robustness, high hydration capacity, favourable rheological and colloidal properties, and excellent biocompatibility. These features position them as strong candidates for controlled release applications in biomedical and pharmaceutical contexts.

## Figures and Tables

**Table 1:** Relative volumes (% *w*/*v*) of CS and GNP mixtures used to obtain NG (S1–S3) samples under different pH conditions.

Sample	pH (CS)	%CS	%GNP
**S1**	3.6	0.3	0.1
**S2**	4.5	0.3	0.1
**S3**	5.5	0.3	0.1

Final volume of each NG-containing solution: 1 mL (CS: 600 µL + GNP: 400 µL).

**Table 2:** Formation rates calculated from the 4P sigmoid fit.

Sample	Phase	(1/t)	R^2^
**S1**	Initial Nucleation	1.01 × 10^−6^ ± 4.76 × 10^−8^	0.9784
**S2**	Initial Nucleation	9.25 × 10^−6^ ± 3.45 × 10^−7^	0.9775
**S3**	Initial Nucleation	2.65 × 10^−5^ ± 1.34 × 10^−6^	0.9682
**S1**	Exponential phase	0.00025 ± 1.56 × 10^−6^	0.9784
**S2**	Exponential Phase	0.0023 ± 9.96 × 10^−5^	0.9775
**S3**	Exponential Phase	0.0066 ± 1.21 × 10^−5^	0.9682

**Table 3:** Parameters derived from the suspension’s characterisation for each CS-GNP combination consideration.

Sample	PDI	ζ-Potential (mV)	G′ (1 Hz)	μ_ap_
**S1**	0.229 ± 0.035 ^a,^*	31.64 ± 1.43 ^a^	4.09 ^a^	0.331 ± 0.045 ^a^
**S2**	0.252 ± 0.024 ^a^	29.35 ± 1.17 ^a^	41.2 ^b^	0.0418 ± 0.0052 ^b^
**S3**	0.274 ± 0.033 ^a^	20.42 ± 0.75 ^c^	18.0 ^c^	0.0336 ± 0.0336 ^c^

* Average values derived from NG PDI, ζ-Potential, G′ evaluated at a fixed frequency (1 Hz) and the apparent viscosity, µ, at the asymptotic portion of the curve. Different letters represent significantly different groups, *p* < 0.05 (Tukey post hoc).

**Table 4:** Time-dependent swelling behaviour of NGs (formulation S2).

Time (h)	Hydrated NG Mass (g)	ESR (%)
**0**	0.010 ± 0.000	0
**4**	0.0167 ± 0.006	66.7
**6**	0.1567 ± 0.015	1466.7
**24**	0.3033 ± 0.010	2933.3
**48**	0.3100 ± 0.012	3000.0
**72**	0.3200 ± 0.011	3100.0
**96**	0.3200 ± 0.012	3100.0
**120**	0.3200 ± 0.012	3100.0

## Data Availability

The data can be shared up on request.

## Supplementary Materials

The following supporting information can be downloaded at: https://www.mdpi.com/article/10.3390/pharmaceutics17070876/s1. Supplementary Figure S1: Visible spectra for single CS and single GNP in the range of 400–750 nm. Supplementary Figure S2. Intensity-based particle size distribution profiles of CS-GNP NG (S1–S3) obtained by DLS, without the application of HIUS. S1 and S2 exhibit multimodal distributions, with S2 showing a dominant peak below 1000 nm. S3 displays a broad monomodal distribution with a peak between 1000 and 10,000 nm, indicating aggregation and transition into the lower micrometre range. Supplementary Figure S3. Stability assessment of NG formulations (S1, S2, and S3) after 21 days of incubation at 37 °C. (A) UV–Vis spectra of NGs showing optical density profiles in the 380–800 nm range; *inset*: magnified view between 550 and 750 nm. (B) Particle size distribution in terms of intensity versus diameter (d.nm) of NG at day 21 obtained by DLS, indicating the presence of both major and minor populations. (C) ζ-potential measurements of S2 at time points t_0_ and t_21_ days; bars with different letters denote statistically significant differences (*p* < 0.01).

## Abbreviations

The following abbreviations were used in this manuscript:

ξ	correlation length
η	viscosity
δ	phase angle
γ	shear rate
ARPE-19	human retinal pigment epithelial cells
CS	chitosan
CS-GNP NG	chitosan–genipin nanogel
d.nm	diameter
DAPI	4’,6-diamidino-2-fenilindol fluorescent probe
DMEM	Dulbecco’s modified Eagle’s medium
DLS	dynamic light scattering
ESR	equilibrium swelling ratio
FBS	foetal bovine serum
FOE	first-order exponential
FTIR	Fourier-transform infrared spectroscopy
G′	viscoelastic storage
G″	loss module
GNP	genipin
HIUS	High-intensity ultrasound
m	power-law exponent
MTT	colorimetric assay used to assess cytotoxicity
NG	nanogel
NP	nanoparticle
L929	mouse fibroblast cell line
PDI	polydispersity index
SAXS	small-angle X-ray scattering
SEM	scanning electron microscopy
SK	sigmoidal kinetics
TEM	transmission electron microscopy
