# Supplementary figures and images for: Perspectives on the pH-Influenced Design of Chitosan–Genipin Nanogels for Cell-Targeted Delivery
Source: Pharmaceutics. 2025 Jul 3;17(7):876. doi: 10.3390/pharmaceutics17070876 (PMC12300693; doi:10.3390/pharmaceutics17070876)

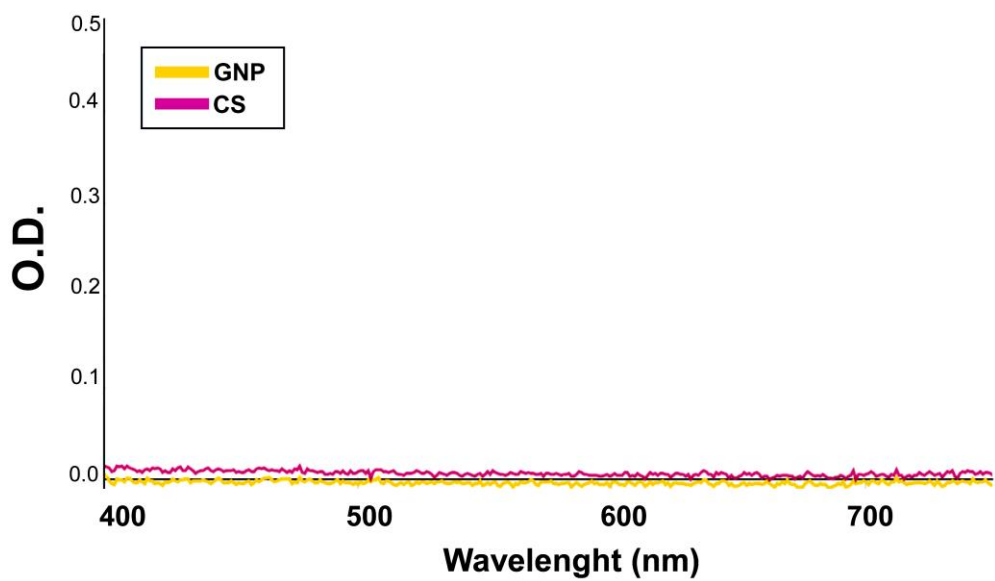

Figure S1.

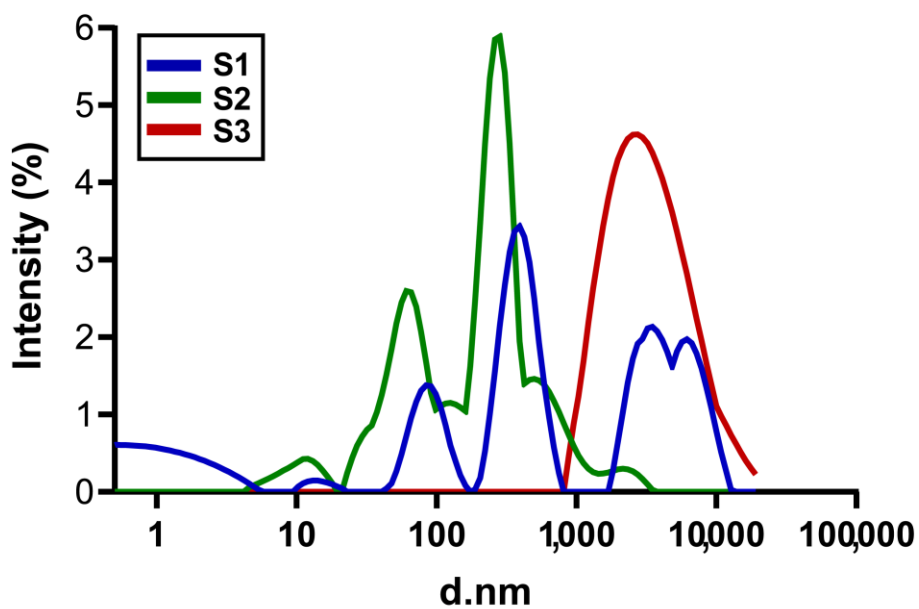

Figure S2.

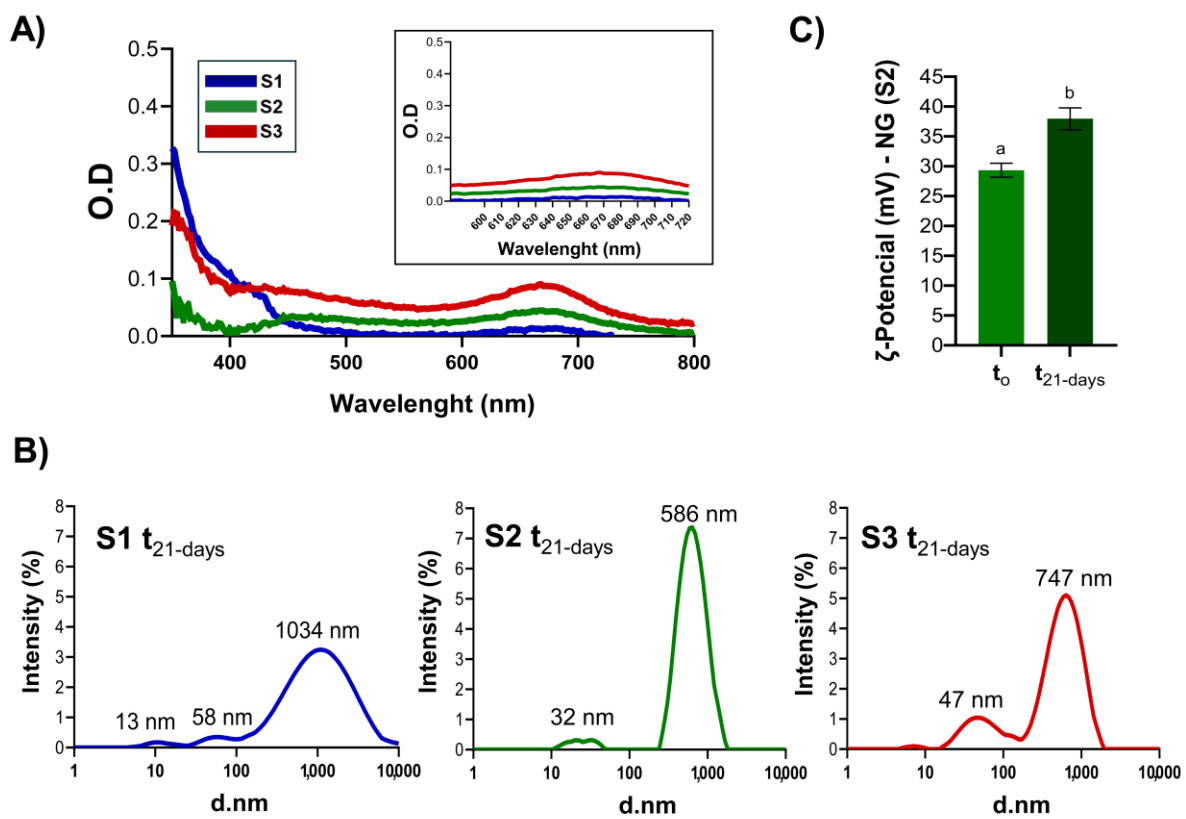

Figure S3.

Supplement: Supplementary file 1 [file pharmaceutics-17-00876-s001.zip › pharmaceutics-3670050-Supplementary.pdf]
